# Complete Genome Sequence of Streptomyces sp. Strain SGAir0924, an Actinobacterium Isolated from Outdoor Air in Singapore

**DOI:** 10.1128/MRA.00899-19

**Published:** 2019-08-29

**Authors:** Anjali Bansal Gupta, Akira Uchida, Rikky W. Purbojati, Anthony Wong, Kavita K. Kushwaha, Alexander Putra, Balakrishnan N. V. Premkrishnan, Cassie E. Heinle, Merrilyn Eng, Vineeth Kodengil Vettath, Ana Carolina M. Junqueira, Daniela I. Drautz-Moses, Stephan C. Schuster

**Affiliations:** a Singapore Centre for Environmental Life Sciences Engineering, Nanyang Technological University, Singapore, Singapore; b Departamento de Genética, Instituto de Biologia, Universidade Federal do Rio de Janeiro, Rio de Janeiro, Brazil; University of Rochester School of Medicine and Dentistry

## Abstract

Streptomyces sp. strain SGAir0924 was isolated from outdoor air collected in Singapore. Its genome was assembled using long reads generated by single-molecule real-time sequencing. The final assembly had one chromosome of 7.65 Mb and three plasmids with an average length of 142 kb. The genome contained 6,825 protein-coding genes, 68 tRNAs, and 18 rRNAs.

## ANNOUNCEMENT

Streptomyces spp. are Gram-positive filamentous bacteria belonging to the phylum Actinobacteria. They are well known for the production of a large variety of natural antibiotics and antifungal and antiparasitic compounds (1). More than 600 species of *Streptomyces* bacteria have been recorded, with the majority of them being recognized as important producers of bioactive compounds (2, 3).

*Streptomyces* spp. primarily inhabit soil and water (4, 5). Here, we report a new strain, *Streptomyces* sp. strain SGAir0924, isolated from outdoor air in Singapore at global positioning system coordinates 1.35°N, 103.68°E. Air was sampled by impaction onto an electrostatic filter attached on an SASS 3100 dry air sampler (Research International, USA). After sampling, the filter was soaked in phosphate-buffered saline containing 0.1% Triton X-100 to suspend the captured particles. The suspension was then plated onto marine agar (Becton, Dickinson, USA), followed by aerobic incubation at 30°C. Colonies were repeatedly picked and plated onto malt extract agar to obtain clonal colonies. For genomic DNA extraction, a single colony was then inoculated in lysogeny broth (Becton, Dickinson) and incubated at 30°C. DNA extraction was performed with the Wizard genomic DNA purification kit (Promega, USA), following the manufacturer’s instructions. The extracted genomic DNA was subjected to library preparation with the SMRTbell template preparation kit 1.0 (Pacific Biosciences, USA), following the manufacturer’s 20-kb template preparation using the BluePippin size selection system protocol. The finished library was then sequenced on the RS II single-molecule long-read sequencing platform (Pacific Biosciences). In total, 45,198 subreads with a mean length of 9,337 bp were obtained.

The sequenced genome of *Streptomyces* sp. strain SGAir0924 was *de novo* assembled using Hierarchical Genome Assembly Process (HGAP) version 3 (6) implemented in the PacBio SMRT Analysis package version 2.3.0 using a seed read length of 500 bp. The consensus assembly generated four contigs with a total length of 8,079,654 bp. This included a linear chromosomal contig (7,653,753 bp, 45.3-fold coverage) and a linear plasmid (377,458 bp, 39.6-fold coverage). Two other plasmids (26,627 bp and 21,816 bp) were able to be circularized using Circlator version 1.1.4 (7). The mean G+C content of the chromosome was 72.6%. The average nucleotide identity (ANI) using the Microbial Species Identifier (MiSI) (8) method revealed Streptomyces silaceus to be the closest relative, with an identity of 83% and an alignment fraction value of 0.22. However, based on the 16S rRNA gene sequence, the closest known species is *Streptomyces* sp. strain NEAU-L11, with 100% identity. As such, strain SGAir0924 can only be identified up to the genus but not the species level. Default parameters were used for all programs unless otherwise specified.

The assembled genome was annotated using the NCBI Prokaryotic Genome Annotation Pipeline (PGAP) version 4.2 (9). A total of 7,166 genes were predicted, including 6,825 protein-coding genes (PCGs), 18 rRNA operons (5S, 16S, and 23S rRNAs), 68 tRNAs, 3 noncoding RNAs, and 252 pseudogenes. Functional annotation with the Rapid Annotations using Subsystems Technology (RAST) server (10–12), using the classic RAST annotation scheme with an option to fix frameshifts, identified a total of 7,054 coding sequences and 85 RNAs covering 445 subsystems. Of those, 49 genes were related to sigmaB stress response regulators. SigmaB is a transcription factor that is switched on under physical stress conditions (13, 14), which may be related to desiccation in air and the survival of this strain under arid conditions. Furthermore, the biosynthetic potential of this strain predicted with antiSMASH (15) resulted in 33 secondary metabolite biosynthetic gene clusters; many of them are related to antimicrobial compounds, such as streptothricin and candicidin.

### Data availability.

The complete genome sequences of *Streptomyces* sp. strain SGAir0924 and its plasmids have been deposited in DDBJ/EMBL/GenBank under accession numbers CP027296, CP027297, CP027298, and CP027299 and in the SRA database under accession number SRR8948646.
